# PSICA: a fast and accurate web service for protein model quality analysis

**DOI:** 10.1093/nar/gkz402

**Published:** 2019-05-25

**Authors:** Wenbo Wang, Zhaoyu Li, Junlin Wang, Dong Xu, Yi Shang

**Affiliations:** 1 Department of Electrical Engineering and Computer Science, University of Missouri, Columbia, MO 65211, USA; 2 Christopher S. Bond Life Sciences Center, University of Missouri, Columbia, MO 65211, USA

## Abstract

This paper presents a new fast and accurate web service for protein model quality analysis, called PSICA (Protein Structural Information Conformity Analysis). It is designed to evaluate how much a tertiary model of a given protein primary sequence conforms to the known protein structures of similar protein sequences, and to evaluate the quality of predicted protein models. PSICA implements the MUfoldQA_S method, an efficient state-of-the-art protein model quality assessment (QA) method. In CASP12, MUfoldQA_S ranked No. 1 in the protein model QA select-20 category in terms of the difference between the predicted and true GDT-TS value of each model. For a given predicted 3D model, PSICA generates (i) predicted global GDT-TS value; (ii) interactive comparison between the model and other known protein structures; (iii) visualization of the predicted local quality of the model; and (iv) JSmol rendering of the model. Additionally, PSICA implements MUfoldQA_C, a new consensus method based on MUfoldQA_S. In CASP12, MUfoldQA_C ranked No. 1 in top 1 model GDT-TS loss on the select-20 QA category and No. 2 in the average difference between the predicted and true GDT-TS value of each model for both select-20 and best-150 QA categories. The PSICA server is freely available at http://qas.wangwb.com/∼wwr34/mufoldqa/index.html.

## INTRODUCTION

The three-dimensional (3D) structure of a protein is essential in studying its functions (1). Computational 3D protein structure prediction is important since experimental methods including X-ray crystallography, electron microscopes and nuclear magnetic resonance (NMR) are all costly and time consuming (2). Predicting 3D structures using computational methods can be much faster and cheaper. However, the accuracy of predicted models can vary greatly for different targets and different prediction methods. Therefore, it is vital to find a reliable method to evaluate the quality of predicted models (3).

Over the past 20 years, many protein model quality assessment (QA) methods have been proposed (4,5). There are two basic approaches: single-model QA methods (6–10) that are able to evaluate a single model's quality, and multi-model QA methods (11,12) that require a pool of models to evaluate the quality of one or some models in the pool. Multi-model QA methods have outperformed single-model QA methods in recent CASPs (4,5,13,14). However, the size and quality of the model pool used by multi-model methods have great impact on their QA results (15). Single-model QA methods use potential functions and/or machine learning. Potential functions include physics-based potential functions and knowledge-based potentials (16). Machine learning has been used to aggregate various potential functions to achieve improved results.

Quasi-single-model QA methods (17) try to combine the advantages of both types of methods. They employ the ‘consensus’ idea from multi-model QA methods, but do not require a pool of models as input. Instead, they generate their own reference models. Quasi-single-model QA methods have achieved good results in recent CASPs, comparable to multi-model QA methods. However, generating a good pool of reference models and making the best use of multiple fragments of known protein structures remain a challenge.

After years of development, state-of-the-art QA methods are becoming complex and difficult for users to implement. The PSICA (Protein Structural Information Conformity Analysis) web service is intended to make some of the best existing QA methods available to the public. It has an intuitive user interface that runs on all mainstream web browsers. Users do not need to run any local software, plug-in, Java Applets or ActiveX.

PSICA is designed to evaluate how much a tertiary model of a given protein primary sequence conforms to the known protein structures of similar protein sequences, and to assess the quality of predicted protein models. PSICA implements the MUfoldQA_S method (18), an efficient state-of-the-art quasi-single-model QA method. In CASP12, MUfoldQA_S ranked No. 1 in the protein model QA select-20 category in terms of the difference between the predicted and true GDT-TS value of each model. MUfoldQA_S calculates quality scores based on templates. which are protein fragments with known 3D structure and sequences similar to the sequence of predicted model. For a given predicted 3D model, PSICA generates (i) predicted global GDT-TS value; (ii) interactive comparison between the model and other related known protein structures; (iii) visualization of the predicted local quality of the model; and (iv) JSmol rendering of the model. The GDT-TS value is a popular indicator of similarity between the two protein 3D structures (in our case, predicted models and observed structures). It is calculated by computing the percentage of corresponding C-alpha atom pairs whose distance falls within the cut-off values of 1, 2, 4 or 8 Å after superimposing the two protein structures, and compute the average of those four percentage values.

Additionally, PSICA implements MUfoldQA_C (18), a multi-model QA method based on MUfoldQA_S. MUfoldQA_C uses MUfoldQA_S results as weights in a consensus approach and let better models contribute more to the final QA result. In CASP12, MUfoldQA_C ranked No. 1 in top 1 model GDT-TS loss in the select-20 QA category and No. 2 in the average difference between the predicted and true GDT-TS value of each model for both select-20 and best-150 QA categories.

In the rest of this paper, after a brief overview of the algorithms, the implementations of the web service will be presented. Then, a detailed description of inputs and results will be given. At last, experimental results on benchmark datasets will be presented to show its advantages over existing servers.

## MATERIALS AND METHODS

### Algorithm overview

MUfoldQA_S is a quasi-single model QA method that predicts the GDT-TS value between a protein model and its native structure. This method calculates model QA score based on the fragments of other known protein structures with similar primary sequences without building full protein models.

The input to MUfoldQA_S is the sequence of amino acids of the target protein (TargetSeq_0) and a predicted model (Decoy_0). Its main steps are as follows:

- Use Blast (19) to query our in-house protein database (20) to find a set of proteins with similar sequences. Blast first uses NR sequence database to generate the checkpoint file and then use this file as input to search PDB sequence database for similar sequences. Let us call the set of similar sequences found by Blast Seq_Blast.
- Sort Seq_Blast according to heuristic score *T = (3-log_10_E)• I• C*, where *I* is the sequence identity, i.e. the extent to which two amino acid sequences have the same residues at the same positions in an alignment, expressed as a percentage; *C* is the ratio of the template length to the target sequence length; and *E* is the *E*-value. The formula combines the three factors together in spite of the different scales. The term *(3-log_10_E)* is designed to return a positive value for all *E* < 1000, which is a good trade-off between covering the majority of the cases and maintaining a good performance.
- Select the top 10 similar sequences (referred to as Seq_Blast_T10) with the highest *T*-scores.
- Repeat Step 1–3 using HHsearch (21) to replace Blast to get another set of similar sequences, referred to as Seq_HH_T10. HHsearch uses a profile HMMs database derived from the PDB sequences to get results.
- Merge Seq_Blast_T10 and Seq_HH_T10 to get Seq_T20, without removing any of these sequences even if some sequences are from the same protein.
- For each C-alpha position *j* on template *k* from the sequence pool Seq_T20, compare its sequence with position *j* on TargetSeq_0 from the input and calculate BLOSUM-based (22) weight *W_kj_*.
- Retrieve the corresponding 3D coordinates of all sequences in Seq_T20 from the PDB database, referred to as Structure_T20.
- Compare the 3D structure of the predicted model (Decoy_0) with each structure in Structure_T20 to calculate GDT-TS value *S_k_*.
- MUfoldQA_S local score *H_j_* at position *j* is the average of *S_k_* weighted by *W_kj_*.
- MUfoldQA_S global score is the simple average of all *H*_j_.

MUfoldQA_C uses the information that MUfoldQA_S generates to improve consensus result. MUfoldQA_C takes in a group of predicted models and runs MUfoldQA_S on each one to generate its local and global scores. Then, a certain number of top models are selected as the reference models according to their global scores. The final QA score for each predicted model is the weighted average of pair-wise GDT-TS value between the model and each reference model, weighted by the local score of MUfoldQA_S. For more details about the database and parameters, please refer to our paper (18).

### Implementation

On PSICA web page, after the user submits a task either through filling out a form on the web page or through an API, the task data will be stored in a queue managed by the server. When a task is executed, the server runs Blast and HHsearch, respectively, to find similar sequences using the target sequence. When a compressed protein model file is submitted, the server unpacks the compressed file to get a set of predicted models. Then, for each model, MUfoldQA_S is run to calculate a global QA score and local QA scores. These results and the original task information are stored in functionality expansion APIs for other programs to use. If MUfoldQA_C add-on is enabled by the user, MUfoldQA_C scores will be computed using MUfoldQA_S data from the APIs. At last, all results are presented and visualized in a user-friendly manner.

The server backend is written in PHP. All frontend user interface and interactive visualization are implemented in HTML5/JavaScript. Neither browser plug-in, Java Applets, nor ActiveX is required. The task scheduler of the server is written in Go without using any SQL statement to eliminate SQL injection. MUfoldQA_S is mainly written in Octave/MATLAB. In addition to third party software Blast and HHsearch, the server also uses TMscore (23,24) to calculate GDT-TS values.

## RESULTS

### Web interface

PSICA requires the input of a protein sequence and one predicted model file in PDB format. Optionally, multiple predicted models can be submitted together in a *.tar.gz file as used in CASP. For users who want to generate their own tarball files, command line instructions for Linux and MacOS and executable files for Windows are provided to simplify the process.

**Figure 1.**
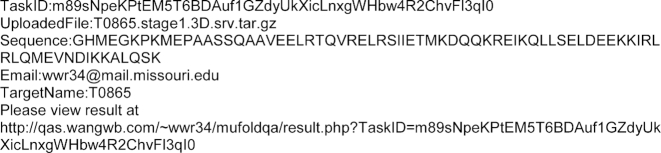
Example of task receipt file in .txt format.

An optional MUfoldQA_C add-on is available, which is useful for QA on a set of predicted models for the same target sequence. The other optional inputs include the Target Name field, which helps the user to remember different submitted tasks, and the Email field, which allows the user to receive a notification email after the task is finished. The notification email includes a text version of the result and a link to the full report. If the user chooses not to provide the email address, the user can still retrieve the results via either the task status page or the task receipt file generated (Figure 1) during the submission process. For people using public computer or simply wish not to receive receipt file, they can use ‘Disable receipt file for this task’ when submitting the task. For users who need a quick demonstration of the server, a sample input with instruction is provided. The sample comes from CASP12 target T0865 stage 1 (select-20) QA task. T0865 is C-terminal coiled-coil domain of CIN85 (PDB code: 2N64). At the length of 75 amino acids, it is one of the shortest targets in CASP 12, and thus minimize the download, upload and computation time needed for the demonstration.

**Figure 2.**
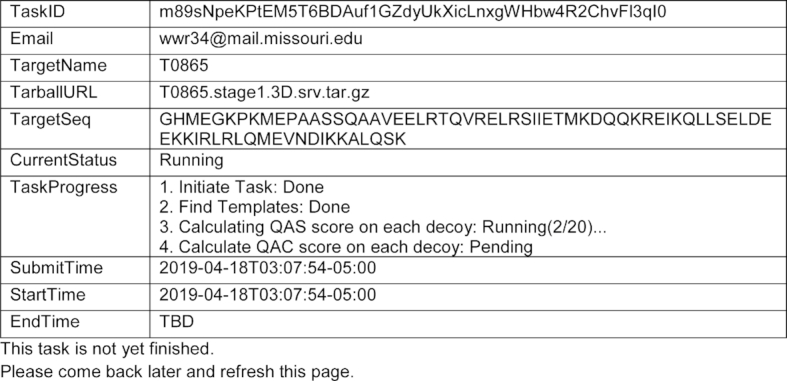
Example of job status web page.

When a user clicks the ‘Submit This Job’ button, the input will first be verified on the frontend and then validated on the backend. If the submission is successful, a task receipt file will be generated to make a local copy of the basic information of the task and the URL to retrieve the result. Then the webpage will automatically redirect to the result page. If the task is still running, this page will show the basic task information (Figure 2). In addition to the information submitted by the user, this page also includes fields indicating if the job is running or waiting in queue (‘CurrentStatus’), the time user submitted this task (‘SubmitTime’), and the time in which this task leaves the waiting queue and starts running (‘StartTime’). When the task is running, the ‘TaskProgress’ section will show a list of subtasks and their statuses.

**Figure 3.**
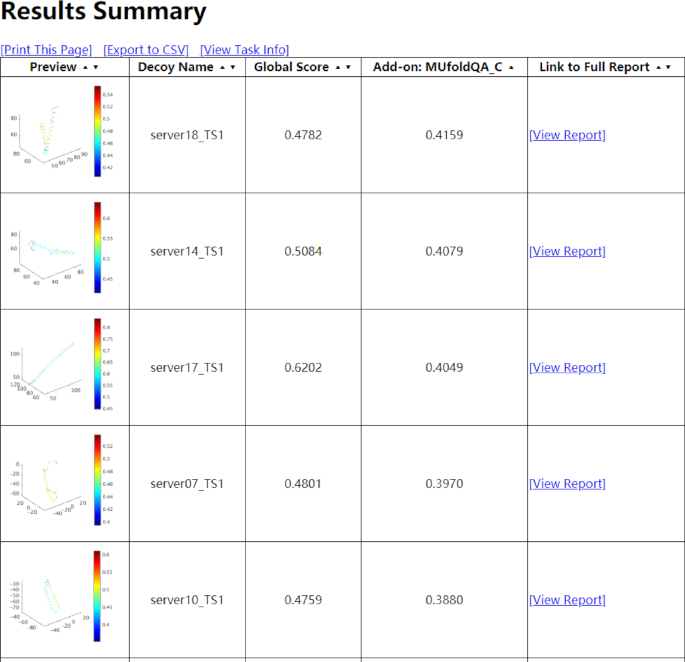
Example of results summary page.

When the task is finished, the result page will display a table showing a summary of the results. From left to right, the columns are visualization of the local quality, name of the predicted model, global score (ranging from 0 to 1, the higher the better), and the link to the full report of this predicted model. The table is sorted by global score by default but can be changed to be sorted by decoy name. If the user has enabled MUfoldQA_C add-on, an additional column of MUfoldQA_C results (ranging from 0 to 1, the higher the better) will be added (Figure 3), and the result will be sorted by MUfoldQA_C scores. A user can export the scores to CSV file by click on ‘[Export to CSV]’ or simply print the page with ‘[Print This Page]’. Below the summary table is the task information table, which recalls some information like when the task starts and finishes, what the sequence is, the max, mean and min sequence identities of the templates.

**Figure 4.**
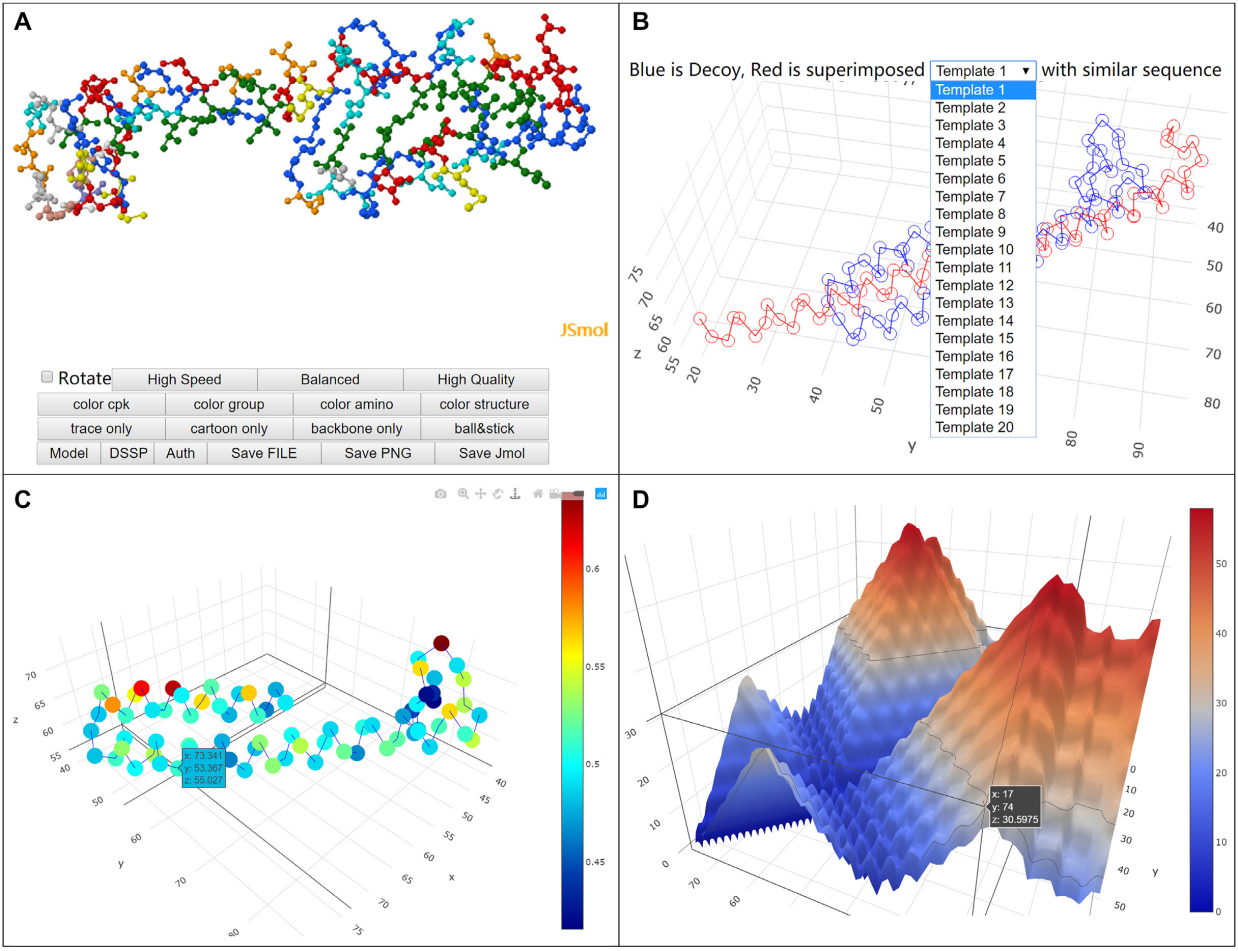
Some of the interactive visualizations in the detailed report page for each of the predicted models. (**A**) JSmol View of the Decoy; (**B**) Comparing Decoy with Different Templates; (**C**) Visualization of Local Quality of the Decoy (Range 0–1, Higher the Better); (**D**) Decoy Distance Matrix.

In the full report, the title will show the model name and its global quality, followed by the interactive JSmol view (Figure 4A) of the model. The JSmol view uses mouse to control rotation and includes widgets to change the way the model is colored and rendered, and to export the model file or take a snapshot of the model.

Next to the JSmol view is the interactive visualization to compare models with templates (Figure 4B). The visualization consists of a pair of 3D C-alpha backbone models superimposed over each other. The blue one represents the predicted model and the red one represents the template. The drop-down menu allows users to select from a pool of 20 templates based on Blast and HHsearch results. In MUfoldQA_S, each one of these templates is superimposed to the predicted model to calculate a similarity score, which is then used to evaluate the quality of the predicted model. More details about each template are also described in a table below the visualization. This table includes Method (Blast/HHsearch), Template ID (which corresponds to the ID in the dropdown menu), Origin (the PDB code), Identity, E-value, Coverage and Score.

Below the template comparison is the visualization of local MUfoldQA_S score (Figure 4C). It is an interactive 3D model of C-alpha backbone in different colors that represent different local qualities. The local quality is calculated for each C-alpha position. This graph shows which section might be ill-folded and helps user to gain insights on how to improve the predicted structure. At last, the visualization of distance matrix (Figure 4D) is provided. Contact map is of great importance in protein structure prediction. It is a 2D binary matrix where each element represents if the distance of two amino acids falls into a cut-off threshold. A contact map could be derived from the distance matrix based on a cut-off threshold. Instead of plotting multiple contact maps that a user might need, the 3D map allows a user to interactively explore how the contour separating contact and non-contact regions changes with different cut-off values by simply moving the mouse.

For developers who want to integrate PSICA into their own software, a script is provided to demonstrate how to interact with PSICA sever, including submitting a task, checking for task status and retrieving the final results in Python, Octave/MATLAB. Since the API for PSICA is simple to use, the script can also be easily adapted to most programming languages that support system calls. In fact, we have already used PSICA in some of our QA methods in CASP 13.

### Benchmark results

To gain an unbiased evaluation of the performance, we have participated in CASP12 under the group name MUfoldQA_S and MUfoldQA_C. CASP (Critical Assessment of Techniques for Protein Structure Prediction) experiment is a world-wide competition held every two years since 1994. It is designed to provide objective evaluation of the state-of-the-art methods for protein structure prediction. In recent CASPs, the QA task consists of two stages. In stage 1, each QA group was given up to 20 selected predicted models (Select-20) ranging from good to bad. In the stage 2, each QA group was given up to 150 top models (Best-150) selected by the naïve consensus algorithm.

#### Accuracy

When blindly tested in CASP12, MUfoldQA_S (scores shown in ‘Global Score’ column in the result summary page in Figure 3) and MUfoldQA_C (scores shown in ‘Add-on: MUfoldQA_C’ column in the result summary page in Figure 3) achieved good results. Table 1 shows the performance comparison between them and other methods in terms of GDT-TS differences between predicted and true values, average over up to 70 targets. All data are from the CASP official website.

**Table 1.** Difference between the predicted and true GDT-TS values of models, average over all targets

CASP12 Select-20					CASP12 Best-150				
GR	CR	Group Name	GN	AD	GR	CR	Group Name	GN	AD
1	1	*MUfoldQA_S*	334	3.602	1	1	**FDUBio**	237	5.173
2	1	**MUfoldQA_C**	318	3.818	2	2	**MUfoldQA_C**	318	5.512
3	2	**Davis-EMAconsensus**	034	5.615	3	1	*ModFOLD6_cor*	360	6.748
4	3	**FDUBio**	237	5.756	4	3	**Davis-EMAconsensus**	034	6.781
5	2	*ModFOLD6*	201	5.883	5	2	*ModFOLD6*	201	7.087
6	3	*ModFOLD6_cor*	360	6.697	6	4	**ModFOLDclust2**	214	7.093
7	4	**ModFOLDclust2**	214	6.878	7	5	**iFold_2**	112	8.373
8	1	Wang4	195	7.021	8	6	**Deepfold-Contact**	219	8.373
9	5	**Wallner**	073	7.272	9	7	**DeepFold-Boom**	223	8.373
10	2	Wang2	206	8.021	10	3	*MUfoldQA_S*	334	8.898
11	3	ProQ3_1_diso	095	8.155	11	8	**naive**	109	9.210
12	4	VoroMQAsr	093	8.275	12	9	**QASproCL**	267	9.664
13	5	ProQ3_1	302	8.449	13	10	**Wallner**	073	9.710
14	6	VoroMQA	224	8.488	14	4	*ModFOLD6_rank*	072	9.754
15	6	**DeepFold-Boom**	223	8.507	15	11	**Pcomb-domain**	411	9.839
16	7	**Deepfold-Contact**	219	8.507	16	1	ProQ3_1	302	10.155
17	8	**naive**	109	8.507	17	2	ProQ3_1_diso	095	10.159
18	9	**iFold_2**	112	8.507	18	3	ProQ3	213	11.418
19	10	**Pcomb-domain**	411	8.560	19	4	MULTICOM-CLUSTER	287	11.445
20	11	**QASproCL**	267	9.107	20	5	*qSVMQA*	120	11.608
		More groups omitted…					More groups omitted…		

GR: Global Ranking, the ranking among all method. CR: Categorical Ranking, the ranking within its QA method category, either single-model QA, quasi-single-model QA or multi-model QA. GN: Group Number: an identification number of the group assigned by the CASP officials. AD: Average difference between the predicted and true GDT-TS value of each model. Different font style represents different type of QA methods: regular for single-model QA method, italic for quasi-single-model QA method, and bold for multi-model QA method. GOAL and COFOLD_QA submitted no more than five predictions, which makes it an unfair comparison when all other group submitted at least 68 predictions, thus removed from ranking.

The lower the GDT-TS differences, the better. Results of the top 20 groups are shown.

For the select-20 QA category, which is similar to practical protein structure prediction situation, where a small number of predictions are generated and their qualities vary greatly, MUfoldQA_S and MUfoldQA_C performed significantly better than other methods in terms of average GDT-TS differences, ranked No. 1 and No. 2, respectively, outperforming the third place by 35.8% and 32.0%. For the Best-150 QA category, MUfoldQA_C ranked No. 2, outperforming the next best, ModFOLD6_cor, by 18.3%. MUfoldQA_S ranked the third place among all single-model and quasi-single-model QA methods. Furthermore, PSICA is tested using the recently released CASP13 dataset. Only 20 targets (out of total 79 valid targets) can be used for QA performance evaluation because both the true GDT-TS of each decoy model of these targets and the performance of each group are available, whereas the other targets are not. PSICA is compared with other publicly available servers/tools and the result is shown in Table 2. The scores of other methods are from the CASP official website. The result shows that MUfoldQA_C outperforms the latest version of ModFOLD7 by 31% in the select-20 QA category and 28% in the Best-150 QA category. MUfoldQA_S also outperforms MULTICOM series and ProQ series in both categories.

**Table 2.** Difference between the predicted and true GDT-TS value of each model for CASP13 20-target subset, average over all targets

CASP13	Select-20		Best-150	
Method	AD	NT	AD	NT
MUfoldQA_C	3.309	20	4.045	20
ModFOLD7	4.788	20	5.645	20
MUfoldQA_S	4.970	20	6.368	20
MULTICOM_CLUSTER	5.041	20	8.235	20
MULTICOM-NOVEL	5.937	20	8.310	20
ProQ3D	7.792	18	9.388	20
ProQ3	10.117	18	11.483	20
ProQ4	16.206	20	14.262	20

AD: Average GDT-TS difference between predicted and true values. NT: Number of targets.

#### Speed

**Figure 5.**
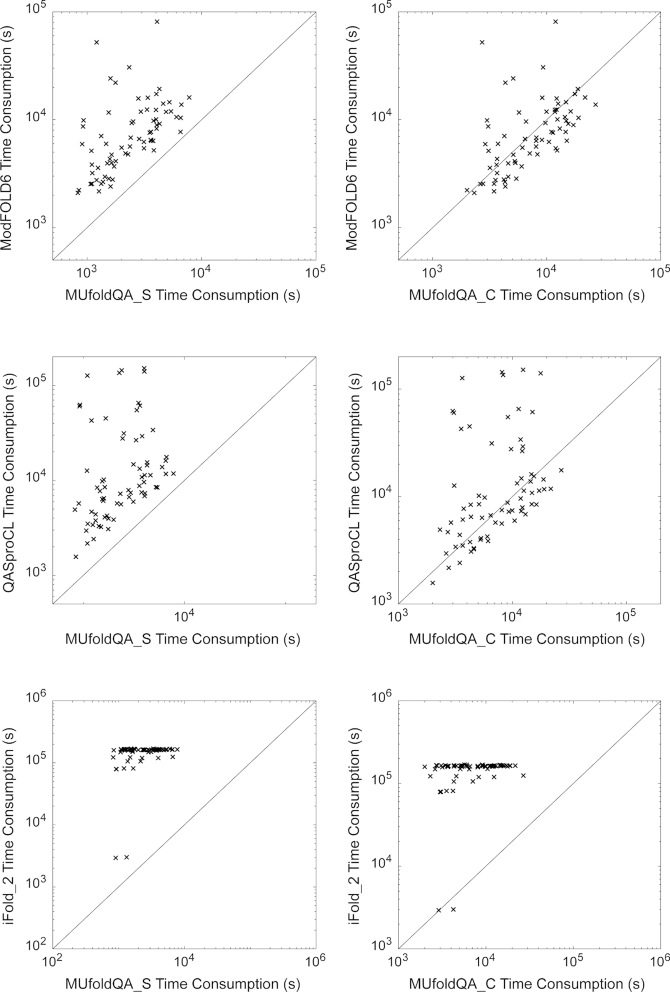
Comparison of execution time between MUfoldQA_S, MUfoldQA_C and some other QA methods (ModFOLD6, iFold_2, QASproCL) on the CASP12 best-150 dataset.

PSICA is much faster than other QA methods, as shown in the comparison of the execution times of PSICA and other methods on the CASP12 best-150 dataset of 70 targets. The execution times of other methods are obtained from the CASP official website, which were calculated as the duration between the timestamp of server received the task and the timestamp of server submitted the result. Figure 5 shows a comparison of execution time between MUfoldQA_S (PSICA with MUfoldQA_C add-on disabled), MUfoldQA_C (PSICA with MUfoldQA_C add-on enabled) and other QA groups. MUfoldQA_S, on average, uses 2,765 s to evaluate a target with 150 decoys. MUfoldQA_C and ModFOLD6 (15) are slower, using 8,694 and 9,961 s, respectively. QASproCL and iFold_2 are much slower, using 23,518 s (7.5 times slower than MUfoldQA_S) and 148,476 s (52.7 times slower than MUfoldQA_S), respectively.

## CONCLUSION

In this paper, we have presented PSICA, a new web service to evaluate predicted protein models by analyzing its conformity to known protein structures. The service is developed based on a top quasi-single-model and a top multi-model QA method in CASP12. It runs faster than other existing servers.

For developers of protein tertiary structure prediction methods, PSICA could be easily integrated into their prediction pipeline. Furthermore, PSICA provides interactive GUIs to visualize varies aspects of the predicted protein model, including interactive comparison between the predicted model and other known protein structures, visualization of the local quality of the predicted model, visualization of its distance matrix and JSmol rendering of the model.

## DATA AVAILABILITY

PSICA is available at http://qas.wangwb.com/∼wwr34/mufoldqa/index.html. This website is free and open to all users and there is no login requirement.
